# Helleborus

**Published:** 1888-08

**Authors:** Edward Adams


### HELLEBORUS.

I feel that I cannot better begin my article upon the above drug and its indication in convulsions than by repeating Hahnemann's description of the effects of this drug, a description I have never seen equaled. He says: "I conclude from various observations that one of the first effects of black Hellebore is a state of stupor, a dullness of the *sensorium commune*, a condition where, with a sight unimpaired, nothing is seen very fully, and the patient does not pay any attention to anything; where, with the hearing perfectly sound, nothing is heard distinctly; where, with properly constituted gustatory organs, everything seems to have lost its genuine taste; where the mind is often or always without ideas; where the past is forgotten or little remembered; where nothing gives one any pleasure; where one's sleep is very light, and a really sound, refreshing sleep is not to be had; and where one desires to work without having the necessary strength or the attention required for it."

Experience has proved how exact were these observations. Mentally we find that the power of the mind over the body is greatly diminished. It is only by a strong effort that the mind is fixed upon the action desired; difficulty in keeping control of two acts at the same time; great depression; weakness of memory; despairing, hopeless, lamenting mood; homesickness; worse from consolation (*cf.* Natr. mur.), does not like to be disturbed; but thinking of symptoms relieves them. (Comp. Camphor.)

The countenance usually expresses the lack of accord between mind and body—stupid, absent. Pressing headache, stupefaction; stupefying headache, with coryza four to eight P. M. (Compare Lyc.)

Rolls head night and day, moaning. Burning heat in head; pale face. Throws head back and from side to side.

Photophobia, without inflammation. Pupils : contracted, dilated ; alternately contracted and dilated. Vacant look, pupils dilated, eyes wide open. Eyeballs turned upward, squinting.

We find convulsive twitchings of the muscles generally.

Automatic motions of one arm and leg. Convulsions, with extreme coldness; of sucklings.

Epilepsy, with consciousness, followed by deep sleep.

Muscles relaxed suddenly.

Eclampsia; a noise or shock shortens the attack.

Except in sleep the arms are continually moving automatically.

Thumb drawn into the palm.

Legs drawn up with every attempt to change her position. Worse at night, from evening until morning. Remission during the day.

Puerperal convulsions. (Urine scanty, albuminous.)

Antidotes: *Camphor*, *Cinchon.*

EDWARD ADAMS.

